# Preliminary exploration of the pathogenesis of patent foramen ovale-related migraine and efficacy analysis of PFO closure

**DOI:** 10.3389/fcvm.2026.1928840

**Published:** 2026-10-20

**Authors:** Mingzhe Ji, Jin Xu, Chengjian Luo, Mengjie Gao, Weiqing Pu, Hua Peng, Jian Xiao

**Affiliations:** 1 Department of Cardiothoracic Surgery, Changzheng Hospital, Naval Medical University, Shanghai, China; 2 Department of Neurology, Changzheng Hospital, Naval Medical University, Shanghai, China

**Keywords:** migraine, patent foramen ovale, proteomics, right-to-left shunt, transcatheter closure

## Abstract

**Background:**

Patent foramen ovale (PFO), a common congenital atrial septal abnormality, can induce intracardiac right-to-left shunt (RLS) and is a potential risk factor for migraine. Currently, there is no unified consensus regarding the therapeutic efficacy and specific molecular mechanisms of PFO closure in the treatment of migraine.

**Methods:**

A single-center retrospective cohort study was conducted, enrolling 107 patients with PFO and migraine, of whom 58 underwent transcatheter PFO closure (observation group) and 49 received standard medical therapy (control group). All patients underwent cardiac imaging and contrast-enhanced transthoracic echocardiography before treatment, with follow-up assessments of headache episodes and scale scores at 1, 3, 6, and 12 months post-treatment. Simultaneously, peripheral arterial blood samples were collected from 5 PFO patients before and after surgery and from 5 healthy controls for plasma proteomic analysis, and changes in arterial partial pressure of oxygen and oxygen saturation were monitored.

**Results:**

The improvements in Visual Analogue Scale (VAS), Headache Impact Test (HIT-6), Migraine Disability Assessment (MIDAS), and Migraine-Specific Quality of Life Questionnaire (MSQ v2.1) scores at each follow-up time point were greater in the observation group than in the control group (all *P* < 0.05). At the 12-month follow-up, the overall treatment effective rate was 94.83% in the observation group, higher than 73.47% in the control group, with a statistically intergroup difference (*P* = 0.003). Stratified efficacy analysis revealed a higher proportion of markedly effective cases and a lower proportion of ineffective cases in the observation group, with statistically differences in the distribution of markedly effective and ineffective cases between groups (*P* < 0.05). Postoperative arterial partial pressure of oxygen and oxygen saturation were markedly improved compared with preoperative levels. Proteomic analysis suggested that the expression levels of inflammation-oxidative stress-related proteins, represented by myeloperoxidase (MPO), lysozyme C (LYZ), and heat shock protein 60 (HSPD1), were downregulated after surgery, and these changes appeared to be associated with the alleviation of hypoxia after elimination of intracardiac shunting.

**Conclusions:**

PFO closure improved migraine symptoms in appropriately selected patients and was associated with downregulation of inflammation-oxidative stress proteins (MPO, LYZ, HSPD1). These findings provide preliminary molecular insights warranting further validation in larger cohorts.

## 1 Introduction

Migraine is one of the most common primary headache disorders, a chronic condition of autonomic nervous system dysfunction characterized by unilateral pulsating headache, often accompanied by nausea and vomiting. The condition tends to recur and persist, severely interfering with patients’ daily work and quality of life, with migraine with aura having a particularly prominent impact on patients’ physical and mental health (1). Epidemiological data show that the prevalence of migraine in adults aged 20–64 years exceeds 10%, with a gender difference, and the male-to-female ratio is approximately 1:3 (2, 3).

Patent foramen ovale (PFO) results from failed fusion of the septum primum and septum secundum after birth and is one of the most common congenital cardiac anomalies. Approximately 20%–30% of adults have incomplete fusion of the foramen ovale, forming a permanent slit-like opening between the atria (4, 5). Normally, left atrial pressure exceeds right atrial pressure, and the flap of the foramen ovale remains closed; however, when right atrial pressure transiently or persistently rises (e.g., during Valsalva maneuver, coughing, or pathological states) and exceeds left atrial pressure, a right-to-left shunt (RLS) can occur, allowing venous blood that has not been filtered by the pulmonary circulation to enter the systemic circulation directly. This abnormal shunt pathway provides an anatomical and physiological basis for the association between PFO and migraine (6).

Clinical epidemiological studies have confirmed that the detection rate of PFO is higher in the migraine population than in the general population, further supporting a close relationship between the two (6–8). In recent years, multiple clinical studies have evaluated the efficacy of transcatheter PFO closure for migraine, with studies specifically targeting patients with migraine with aura showing that the procedure can effectively reduce headache frequency and severity (9, 10). However, several RCTs (MIST, PRIMA, PREMIUM) failed to meet their primary endpoints, leaving the clinical application of PFO closure in migraine controversial (1, 4, 5, 11). These trials shared a common limitation: overly broad inclusion criteria without molecular stratification. For example, PREMIUM showed greater benefit in aura patients by subgroup analysis, and MIST did not stratify by RLS grade—both suggesting that biological heterogeneity confound treatment effects not captured by imaging alone.

Moreover, the specific molecular mechanisms by which PFO triggers migraine remain elusive. Existing hypotheses—paradoxical embolism, escape of vasoactive substances from pulmonary inactivation, genetic susceptibility, and impaired cerebral autoregulation—predominantly focus on hemodynamic or neurovascular aspects and fail to systematically explain at the molecular level why some patients respond dramatically to closure while others do not (12–14). To address this, the present study employed a single-center retrospective cohort design enrolling 107 patients with PFO and migraine, comparing the clinical efficacy of transcatheter PFO closure with standard medical therapy. Concurrently, plasma proteomics was applied to systematically screen for changes in plasma protein expression profiles before and after closure, aiming to explore the potential molecular mechanisms of PFO-related migraine and to provide a reference for precision patient selection.

## 2 Materials and methods

### 2.1 Study population

This was a single-center retrospective cohort study enrolling patients with PFO and migraine hospitalized in the Department of Cardiovascular Surgery at Shanghai Changzheng Hospital between January 2023 and January 2026. All patients were diagnosed with PFO and confirmed to have RLS by transthoracic echocardiography (TTE), transesophageal echocardiography (TEE), and contrast-enhanced transthoracic echocardiography (cTTE). All patients underwent cranial MRI before the procedure to exclude other intracranial lesions that cause migraine. The study protocol was approved by the Ethics Committee of Shanghai Changzheng Hospital, and informed consent was waived due to the retrospective nature of the study.

#### 2.1.1 Inclusion and exclusion criteria

Inclusion criteria:

1. Observation group: ① Diagnosed with migraine according to the International Classification of Headache Disorders, 3rd edition (ICHD-3) criteria; ② Diagnosed with PFO and RLS by imaging; ③ Underwent transcatheter PFO closure; ④ Aged 18–75 years; ⑤ No history of severe heart disease, neurological disease, or other severe systemic diseases.
2. Control group: ① Diagnosed with migraine (ICHD-3 criteria); ② Diagnosed with PFO and RLS by imaging; ③ Received standard medical therapy without PFO closure; ④ Aged 18–60 years; ⑤ No history of severe heart disease, neurological disease, or other severe systemic diseases.

Exclusion criteria:

(1) Pregnant or lactating women; (2) Concomitant severe cardiovascular disease or other conditions requiring urgent treatment; (3) Contraindications to study medications or occluder materials; (4) Incomplete clinical data or loss to follow-up.

#### 2.1.2 Grouping

A total of 107 eligible patients were ultimately enrolled and divided into two groups according to treatment strategy: 58 patients in the observation group who underwent transcatheter PFO closure, and 49 patients in the control group who received standard medical therapy.

### 2.2 Clinical data collection

#### 2.2.1 Baseline data

Demographic characteristics (age, sex), migraine type (aura presence, attack frequency, duration, accompanying symptoms), duration of migraine history, and prior treatment history were collected for all patients.

#### 2.2.2 Imaging assessment

1. Contrast-enhanced echocardiography and transesophageal echocardiography: All patients underwent contrast-enhanced TTE (cTTE), and transesophageal echocardiography (TEE) for PFO diagnosis and RLS grading. RLS grade was assessed by cTTE using a standard microbubble grading system (Grade 0–3) at rest and after Valsalva maneuver. TEE findings were used to confirm RLS and evaluate the anatomical suitability for device closure.
2. Contrast-enhanced transcranial Doppler (TCD): Performed in all patients as a complementary tool for RLS detection and grading, with monitoring of microembolic signals in the middle cerebral artery.
3. Cranial MRI: Exclusion of intracranial space-occupying lesions, vascular malformations, and other brain lesions that cause secondary headache.

#### 2.2.3 Clinical efficacy assessment

All patients were followed up via outpatient visits, hospitalization, or telephone at 1, 3, 6, and 12 months postoperatively. Baseline data were collected before the procedure. Contrast-enhanced transthoracic echocardiography (cTTE) was routinely performed at 1 month postoperatively to confirm occluder position and evaluate residual shunt. The following indicators were recorded at each follow-up time point:

1. Headache episodes: Monthly migraine days, attack frequency, and headache severity assessed by Visual Analogue Scale (VAS).
2. Scale assessments:
  Headache Impact Test (HIT-6): Assessment of the impact of headache on quality of life;
  Migraine Disability Assessment (MIDAS): Assessment of disability caused by migraine;
  Migraine-Specific Quality of Life Questionnaire (MSQ v2.1): Assessment of the impact of migraine on patients’ daily functioning;
  Beck Depression Inventory-II (BDI-II): Assessment of patients’ emotional state and depression severity.

#### 2.2.4 Efficacy criteria

Efficacy was determined at the 12-month follow-up based primarily on changes in VAS score:

Markedly effective: VAS score decreased by ≥5 points from baseline, or postoperative VAS score was 0;

Effective: VAS score decreased from baseline but by <5 points (and did not reach 0);

Ineffective: VAS score did not decrease or increased from baseline.

$$\begin{array}{rl} & \mathrm{Overall}\;\mathrm{treatment}\;\mathrm{effective}\;\mathrm{rate}\\ & =(\mathrm{number}\;\mathrm{of}\;\mathrm{markedly}\;\mathrm{effective}\;\mathrm{cases}\\ & +\mathrm{number}\;\mathrm{of}\;\mathrm{effective}\;\mathrm{cases})\;/\;\mathrm{total}\;\mathrm{cases}\times 100\text{\%}. \end{array}$$

### 2.3 Plasma sample collection and proteomic analysis

#### 2.3.1 Experimental grouping

Five PFO-migraine patients who achieved complete migraine resolution after closure (preoperative VAS ≥ 5 and postoperative VAS = 0) were randomly selected from the observation group to form the experimental group. These five patients included 3 females and 2 males, aged 34–58 years; all presented with moderate-to-large RLS and migraine with aura, and their demographic characteristics were comparable to those of the overall observation group. Five age- and sex-matched healthy volunteers were recruited as the healthy control group.

#### 2.3.2 Blood sample collection

1. Experimental group: Peripheral venous blood and radial arterial blood (5 mL each) were collected before and 1 month after surgery, and left atrial blood (5 mL) was collected during PFO closure, all placed in EDTA anticoagulant tubes.
2. Healthy control group: Peripheral venous blood and radial arterial blood (5 mL each) were collected and placed in EDTA anticoagulant tubes.

All blood samples were centrifuged at 3000 r/min for 15 min at 4 °C, and plasma was separated and aliquoted into 1.5 mL nuclease-free Eppendorf tubes and stored at −80 °C until use.

#### 2.3.3 Proteomic analysis

Tandem mass tag (TMT) labeling combined with liquid chromatography-tandem mass spectrometry (LC-MS/MS) was used for proteomic analysis. The specific steps were as follows:

1. Plasma proteins were digested with trypsin and labeled with TMT reagents;
2. Labeled peptides were fractionated by high-pH reversed-phase chromatography and then subjected to LC-MS/MS for data acquisition;
3. Raw mass spectrometry data were searched and quantified against databases using Proteome Discoverer or MaxQuant software;
4. Differentially expressed proteins were screened with thresholds of |log₂FC| ≥ 1 and *P* < 0.05, and Gene Ontology (GO) functional annotation and Kyoto Encyclopedia of Genes and Genomes (KEGG) pathway enrichment analysis were performed on the differentially expressed proteins.

#### 2.3.4 Enzyme-linked immunosorbent assay (ELISA) validation

Proteins with differential expression and potential clinical relevance (such as inflammatory cytokines and neurotransmitter-related proteins) identified by proteomic analysis were selected for quantitative detection in plasma samples using ELISA kits to validate the reliability of the proteomic results. All operations were performed strictly according to the kit instructions.

### 2.4 Blood oxygen monitoring

Arterial blood was collected before and 1 month after surgery, and arterial partial pressure of oxygen (PaO₂) and arterial oxygen saturation (SaO₂) were measured using an automatic blood gas analyzer to compare preoperative and postoperative changes.

### 2.5 Statistical methods

SPSS 27.0 statistical software was used for data analysis. Normality was tested using the Shapiro–Wilk test.

Normally distributed continuous data are presented as mean ± standard deviation (x¯ ± s), with intergroup comparisons using independent samples t-test and intragroup preoperative-to-postoperative comparisons using paired t-test.

Non-normally distributed continuous data are presented as median (interquartile range) [M (Q₁, Q₃)], with intergroup comparisons using the Mann–Whitney U test.

Multivariate linear regression was used to adjust for age, sex, baseline RLS grade, and migraine subtype in analyzing the primary outcome measures.

Post-hoc subgroup analysis stratified by baseline RLS grade was performed using independent samples t-test.

Intragroup comparisons across different time points for continuous data were performed using repeated-measures ANOVA, with F values reported.

Categorical data are presented as numbers (percentages) [n (%)], with intergroup comparisons using the *χ*^2^ test, and Fisher’s exact test when the expected frequency was <5.

A *P*-value <0.05 was considered statistically significant.

## 3 Results

### 3.1 Comparison of overall clinical efficacy between the observation and control groups

#### 3.1.1 Follow-up and baseline data

A total of 107 patients with PFO and migraine were enrolled, including 58 in the observation group and 49 in the control group. The follow-up rates were 100% (107/107) at 1 month, 100% (107/107) at 3 months, 100% (107/107) at 6 months, 98.13% (105/107) at 9 months, and 98.13% (105/107) at 12 months postoperatively. There were no statistically differences between the two groups in sex distribution, age, or history of comorbidities (*P* > 0.05), indicating comparability (Table 1).

**Table 1:** Comparison of baseline characteristics between the two groups ($\bar{x}$±s).

Characteristics	Observation group (*n* = 58)	Control group (*n* = 49)	Test statistic	*P* value
Sex, male/female, *n*	17/41	16/33	*χ*^2^ = 0.14	0.709
Age, years, mean ± SD	42.43 ± 15.42	53.24 ± 12.10	*t* = 1.55	0.142
Migraine with aura, *n* (%)	38 (65.5%)	32 (65.3%)	*χ*^2^ = 0.01	0.942
Migraine frequency, attacks/month	4.62 ± 2.18	4.38 ± 2.05	*t* = 0.58	0.563
Baseline VAS score	6.20 ± 2.44	4.59 ± 1.50	*t* = 3.98	<0.001
RLS grade (small/moderate/large), *n*	0/20/38	37/12/0	*χ*^2^ = 76.52	<0.001
Photophobia and phonophobia, *n*	15	11	*χ*^2^ = 0.17	0.682
Nausea and vomiting, *n*	14	9	*χ*^2^ = 0.52	0.469
Syncope, *n*	1	0	*χ*^2^ = 0.85	0.356
Previous stroke or epilepsy, *n*	3	2	*χ*^2^ = 0.07	0.790
Arrhythmia, *n*	10	8	*χ*^2^ = 0.02	0.900

Data are presented as mean ± SD for continuous variables and as *n* (%) for categorical variables. For continuous variables, the test statistic is the *t*-value from the independent samples t-test; for categorical variables, the test statistic is the *χ*^2^ value from the chi-square test.

RLS, right-to-left shunt; VAS, visual analogue scale.

Preoperative contrast-enhanced echocardiography showed 38 cases of large RLS and 20 cases of moderate RLS in the observation group, and 12 cases of moderate RLS and 37 cases of small RLS in the control group (the difference in RLS grade distribution between groups was related to treatment selection, which is an inherent feature of retrospective studies).

#### 3.1.2 Within-group comparison of headache-related scales in the observation group

VAS, HIT-6, MSQ v2.1, MIDAS, and BDI-II scores at all postoperative time points were lower than preoperative scores in the observation group, with statistically differences (*P* < 0.001) (Table 2).

**Table 2:** Comparison of scale scores before and after surgery in the observation group ($\bar{x}$ ± s, *n* = 58).

Time point	VAS	HIT-6	MSQ2.1	MIDAS	BDI-II
Preoperative	6.20 ± 2.44	62.95 ± 9.83	50.17 ± 16.16	13.81 ± 20.25	11.14 ± 7.61
1 week postoperative	2.95 ± 2.69	48.53 ± 12.00	31.67 ± 16.48	6.09 ± 13.01	5.03 ± 7.46
1 month postoperative	3.10 ± 2.76	49.24 ± 12.28	32.38 ± 15.52	6.03 ± 13.14	4.98 ± 7.35
3 months postoperative	2.91 ± 2.53	48.00 ± 11.76	31.10 ± 14.73	5.90 ± 11.86	4.81 ± 7.29
6 months postoperative	2.69 ± 2.42	48.00 ± 11.76	29.98 ± 14.06	5.43 ± 11.07	4.76 ± 7.41
9 months postoperative	2.77 ± 2.45	46.59 ± 11.46	29.68 ± 14.02	5.29 ± 10.38	5.00 ± 7.60
1 year postoperative	2.79 ± 2.46	46.16 ± 11.85	29.29 ± 14.17	5.02 ± 9.67	5.11 ± 7.95
F	50.907	52.885	64.297	21.685	120.377
P	<0.001	<0.001	<0.001	<0.001	<0.001

VAS, visual analogue scale; HIT-6, Headache Impact Test; MSQ v2.1, Migraine-Specific Quality of Life Questionnaire version 2.1; MIDAS, Migraine Disability Assessment; BDI-II, Beck Depression Inventory-II. F value is the statistic of repeated-measures ANOVA.

#### 3.1.3 Changes in blood oxygen parameters

PaO₂ and SaO₂ at 1 month postoperatively were improved compared with preoperative levels in the observation group, with statistically differences (*P* < 0.01) (Table 3).

**Table 3:** Comparison of blood gas parameters before and 1 month after surgery in the observation group ($\bar{x}$ ± s, *n* = 58).

Time point	PaO₂ (mmHg)	SaO₂ (%)
Preoperative	82.86 ± 11.68	95.28 ± 2.17
1 month postoperative	85.84 ± 10.91	96.69 ± 1.44
t	−6.69	−7.89
P	<0.01	<0.01

PaO₂, arterial partial pressure of oxygen; SaO₂, arterial oxygen saturation.

#### 3.1.4 Postoperative complications

Among the 58 patients who underwent transcatheter PFO closure in the observation group, 8 (13.8%) developed palpitations after activity, and 2 (3.4%) developed chest tightness postoperatively; Postoperative contrast-enhanced transthoracic echocardiography (cTTE) was performed in all 58 patients at 1 month after the procedure, which confirmed complete closure of the foramen ovale with no evidence of residual right-to-left shunt in any patient. All of these patients had normal electrocardiogram and echocardiography findings at 1-year follow-up. New-onset atrial fibrillation occurred in 2 patients (3.4%), both of whom spontaneously reverted to sinus rhythm with no recurrence of palpitations during the 12-month study follow-up; routine clinical follow-up data extending to 2 years also showed normal electrocardiograms in both patients. No serious complications such as cardiac tamponade, device migration or embolization, thrombosis, infection, or arteriovenous fistula occurred in the entire group (Table 4).

**Table 4:** Postoperative complications in the observation group.

Complication	Cases	Incidence	Follow-up outcome
Palpitations after activity	8	13.80%	Normal ECG and echocardiography at 1 year
Chest tightness	2	3.45%	Normal ECG and echocardiography at 1 year
New-onset atrial fibrillation	2	3.45%	Spontaneous reversion to sinus rhythm, no recurrence during 12-month follow-up; routine clinical follow-up at 2 years also showed normal ECG.
Serious complications (cardiac tamponade, device migration/embolization, thrombosis, infection, arteriovenous fistula)	0	0.00%	No related adverse events

#### 3.1.5 Intergroup comparison of scale score improvements

Because baseline scores on some scales differed between the two groups (VAS, HIT-6, MSQ v2.1, and BDI-II baseline values showed statistically significant intergroup differences, *P* < 0.05), the pre-to-post treatment differences (i.e., improvement values) were used for intergroup efficacy comparison.

##### 3.1.5.1 VAS score

The preoperative VAS score was 6.20 ± 2.44 in the observation group and 4.59 ± 1.50 in the control group (*P* < 0.01). Using improvement values for intergroup comparison, the observation group showed greater VAS improvement at all follow-up time points than the control group (*P* < 0.001), with a marked decrease observed as early as 1 week postoperatively that persisted through 1 year (Table 5).

**Table 5:** Comparison of VAS improvement between the two groups ($\bar{x}$ ± s).

Group	1 week	1 month	3 months	6 months	9 months	1 year
Observation (*n* = 58)	3.28 ± 2.10	3.12 ± 2.12	3.31 ± 2.19	3.53 ± 2.21	3.50 ± 2.26	3.48 ± 2.19
Control (*n* = 49)	0.92 ± 0.91	0.94 ± 0.92	0.96 ± 0.94	0.94 ± 0.94	0.94 ± 0.94	0.94 ± 0.94
t	7.73	7.09	7.42	8.11	7.72	7.87
P	<0.001	<0.001	<0.001	<0.001	<0.001	<0.001

VAS, visual analogue scale; higher scores indicate greater pain severity. Improvement value = preoperative score−postoperative score; negative values indicate worsening of pain compared with baseline.

##### 3.1.5.2 HIT-6 score

The preoperative HIT-6 score was 62.95 ± 9.83 in the observation group and 52.33 ± 9.75 in the control group (*P* < 0.01). The observation group showed greater HIT-6 improvement at all follow-up time points than the control group (*P* < 0.001) (Table 6).

**Table 6:** Comparison of HIT-6 improvement between the two groups ($\bar{x}$ ± s).

Group	1 week	1 month	3 months	6 months	9 months	1 year
Observation (*n* = 58)	14.41 ± 10.32	13.71 ± 10.49	14.95 ± 9.81	14.95 ± 9.81	16.39 ± 10.09	16.82 ± 10.74
Control (*n* = 49)	2.10 ± 2.80	2.06 ± 2.76	2.08 ± 2.75	2.08 ± 2.75	2.02 ± 2.74	2.02 ± 2.74
t	8.72	8.13	9.55	9.55	10.22	9.94
P	<0.001	<0.001	<0.001	<0.001	<0.001	<0.001

HIT-6, Headache Impact Test; higher scores indicate greater impact of headache on quality of life. Improvement value = postoperative score−preoperative score; negative values indicate worsening of quality-of-life impact compared with baseline.

##### 3.1.5.3 MSQ v2.1 score

The preoperative MSQ v2.1 score was 50.17 ± 16.16 in the observation group and 44.35 ± 10.18 in the control group (*P* < 0.05). The observation group showed greater MSQ v2.1 improvement at all follow-up time points than the control group (*P* < 0.001) (Table 7).

**Table 7:** Comparison of MSQ v2.1 improvement between the two groups ($\bar{x}$ ± s).

Group	1 week	1 month	3 months	6 months	9 months	1 year
Observation (*n* = 58)	18.50 ± 15.42	17.79 ± 13.96	19.07 ± 12.83	20.19 ± 13.76	20.34 ± 14.29	20.73 ± 14.85
Control (*n* = 49)	1.10 ± 2.01	1.20 ± 2.20	1.29 ± 2.29	1.31 ± 2.30	1.32 ± 2.17	1.32 ± 2.17
t	8.51	8.92	10.36	10.28	9.83	9.66
P	<0.001	<0.001	<0.001	<0.001	<0.001	<0.001

MSQ v2.1, Migraine-Specific Quality of Life Questionnaire version 2.1; higher scores indicate greater impairment of quality of life. Improvement value = postoperative score−preoperative score; negative values indicate worsening of quality-of-life impairment compared with baseline.

##### 3.1.5.4 MIDAS score

The preoperative MIDAS score was 13.81 ± 20.25 in the observation group and 9.47 ± 10.07 in the control group (*P* > 0.05). The observation group showed greater MIDAS improvement at all follow-up time points than the control group (*P* < 0.001) (Table 8).

**Table 8:** Comparison of MIDAS improvement between the two groups ($\bar{x}$ ± s).

Group	1 week	1 month	3 months	6 months	9 months	1 year
Observation (*n* = 58)	7.72 ± 12.94	7.78 ± 11.34	7.91 ± 10.92	8.38 ± 11.20	8.95 ± 11.52	9.21 ± 12.03
Control (*n* = 49)	0.76 ± 1.69	0.71 ± 1.57	0.71 ± 1.58	0.71 ± 1.58	0.71 ± 1.58	0.71 ± 1.58
t	4.06	4.32	4.57	5.15	5.29	5.23
P	<0.001	<0.001	<0.001	<0.001	<0.001	<0.001

MIDAS, Migraine Disability Assessment; higher scores indicate greater disability caused by migraine. Improvement value = postoperative score−preoperative score; negative values indicate reduction in disability compared with baseline.

##### 3.1.5.5 BDI-II score

The preoperative BDI-II score was 11.14 ± 7.61 in the observation group and 6.27 ± 3.73 in the control group (*P* < 0.01). The observation group showed greater BDI-II improvement at all follow-up time points than the control group (*P* < 0.001) (Table 9).

**Table 9:** Comparison of BDI-II improvement between the two groups ($\bar{x}$ ± s).

Group	1 week	1 month	3 months	6 months	9 months	1 year
Observation (*n* = 58)	6.10 ± 3.69	6.16 ± 3.53	6.33 ± 3.38	6.38 ± 3.43	6.43 ± 3.31	6.32 ± 3.47
Control (*n* = 49)	0.53 ± 0.87	0.53 ± 0.84	0.57 ± 0.94	0.57 ± 0.94	0.57 ± 0.94	0.57 ± 0.94
t	11.13	11.75	12.41	12.37	12.67	11.93
P	<0.001	<0.001	<0.001	<0.001	<0.001	<0.001

BDI-II, Beck Depression Inventory-II; higher scores indicate greater depression severity. Improvement value = postoperative score -preoperative score; negative values indicate worsening of depression compared with baseline.

#### 3.1.6 Comparison of clinical efficacy

At the 12-month follow-up, the overall treatment effective rate was 94.83% (55/58) in the observation group, higher than 73.47% (36/49) in the control group (*χ*^2^ = 4.384, *P* = 0.003). Stratified analysis showed that the markedly effective rate was 63.79% (37/58) in the observation group versus 20.41% (10/49) in the control group, and the ineffective rate was 5.17% (3/58) versus 26.53% (13/49), with both differences reaching statistical significance (*P* < 0.05). The difference in the effective rate (31.03% [18/58] vs. 53.06% [26/49]) was not statistically (*P* = 0.421) (Table 10).

**Table 10:** Comparison of clinical efficacy at 12-month follow-up.

Group	Markedly effective	Effective	Ineffective	Overall effective
Observation (*n* = 58)	37 (63.79)	18 (31.03)	3 (5.17)	55 (94.83)
Control (*n* = 49)	10 (20.41)	26 (53.06)	13 (26.53)	36 (73.47)
*χ* ^2^	1.54	0.16	3.36	4.38
P	0.032	0.421	0.026	0.003

Overall effective rate = (markedly effective cases + effective cases)/total cases × 100%; efficacy criteria as described in Section 2.2.4.

#### 3.1.7 Multivariate regression analysis for confounding adjustment

To account for baseline differences between the two groups, multivariate linear regression analysis was performed with the 12-month VAS improvement value as the dependent variable, adjusting for age, sex, baseline RLS grade, and migraine subtype. After adjustment, PFO closure remained associated with greater VAS improvement (*β* = 2.86, 95% CI: 1.92–3.80, *P* < 0.001), independent of RLS grade and other covariates. These results suggest that the observed treatment effect cannot be fully attributed to baseline imbalances alone.

#### 3.1.8 Subgroup analysis by baseline RLS grade

To evaluate whether baseline RLS grade influenced the efficacy of PFO closure, we performed a subgroup analysis comparing the 12-month VAS improvement between patients with large RLS (Grade 3, *n* = 38) and those with moderate RLS (Grade 2, *n* = 20) within the observation group. Patients with large RLS showed greater VAS improvement compared to those with moderate RLS (large RLS: 4.12 ± 2.08 vs. moderate RLS: 2.86 ± 1.95, *P* = 0.032), suggesting that patients with larger baseline shunts experience a greater degree of migraine relief after PFO closure. However, given the limited sample size in the moderate RLS subgroup, this finding should be interpreted with caution and requires confirmation in larger studies.

### 3.2 Proteomic and metabolomic analysis

#### 3.2.1 Protein identification and quantification

TMT labeling combined with LC-MS/MS was used to detect plasma proteins in 5 patients with PFO and migraine (preoperative radial arterial blood OE1 and postoperative radial arterial blood OE2) and 5 healthy controls (radial arterial blood NC). A total of approximately 790 proteins were identified (Figure 1).

**Figure 1:**
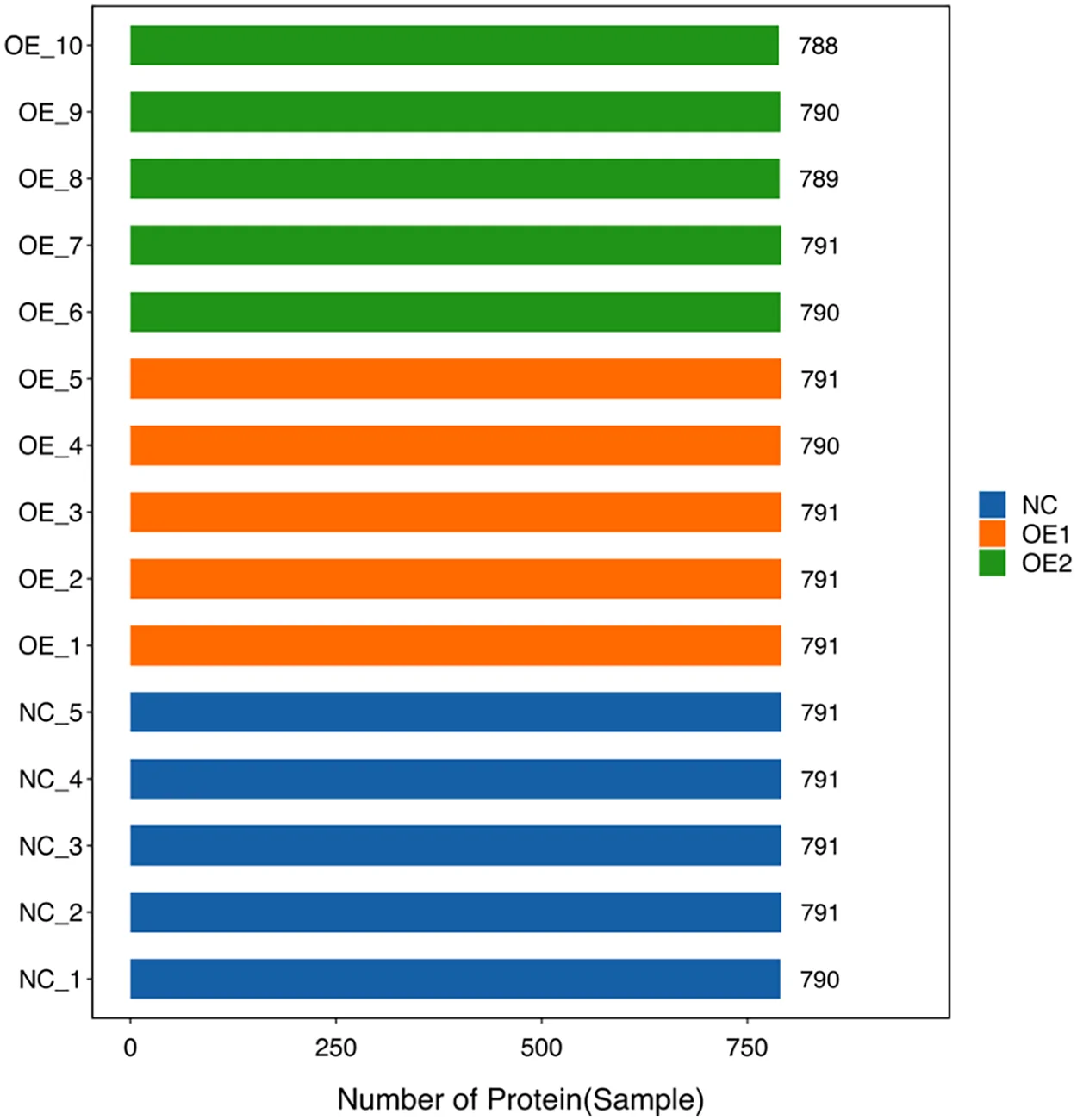
Protein identification and quantification statistics. OE1: preoperative arterial blood (orange); OE2: 1-month postoperative arterial blood (green); NC: control arterial blood (blue).

#### 3.2.2 Principal component analysis

Principal component analysis (PCA) showed that PFO patients with migraine (OE group) and healthy controls (NC group) displayed a clear separation along PC1 (30.1%) and PC2 (21%), indicating differences in protein expression profiles between the two groups. OE1 (preoperative) and OE2 (postoperative) also showed a certain degree of separation in the PCA plot (Figure 2).

**Figure 2:**
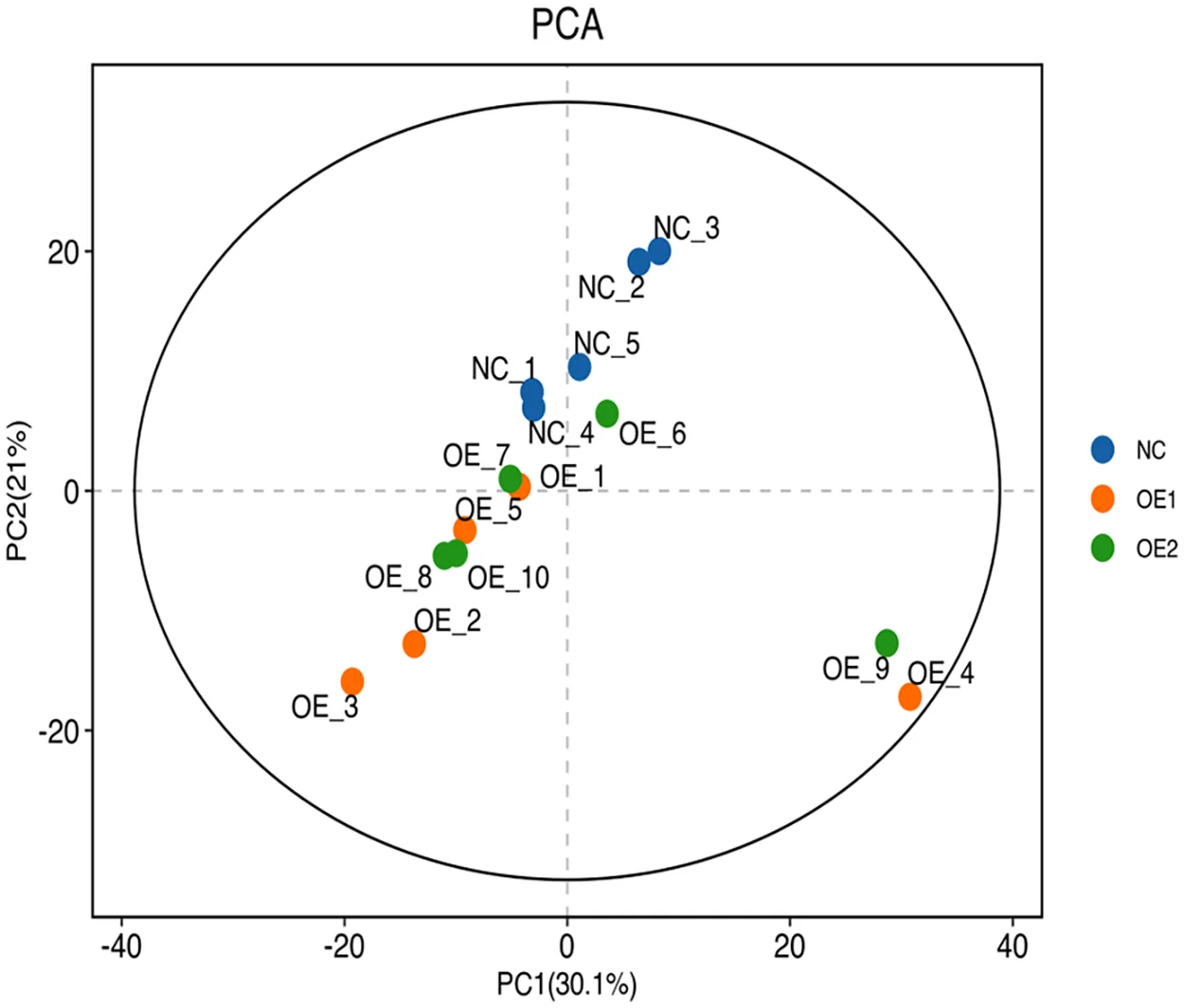
Principal component analysis (PCA). PCA scatter plot showing distribution of NC (blue), OE1 (orange), and OE2 (green) along PC1 (30.1%) and PC2 (21%).

#### 3.2.3 Differential protein screening

With thresholds of |log₂FC| ≥ 0.263 (FC ≥ 1.2 for upregulation, FC ≤ 1/1.2 for downregulation) and *P* < 0.05:

1. OE1 vs. NC: 146 differentially expressed proteins were identified, including 32 upregulated and 114 downregulated (Figure 3).
2. OE2 vs. NC: 114 differentially expressed proteins were identified, including 31 upregulated and 83 downregulated (Figures 3, 4).
3. OE1 vs. OE2: 18 differentially expressed proteins were identified, including 13 upregulated and 5 downregulated (Figures 3, 4).

**Figure 3:**
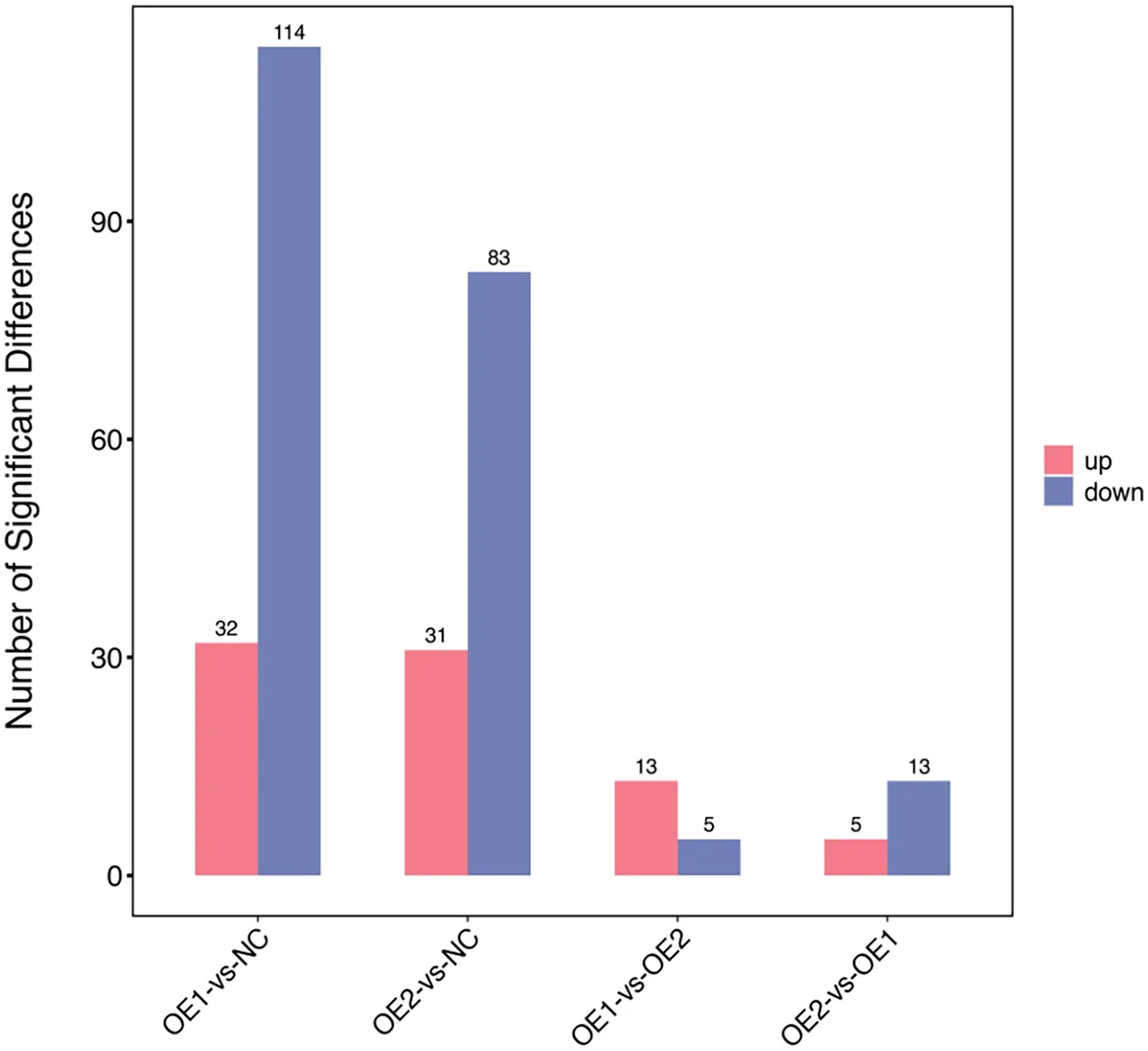
Bar chart of differentially expressed proteins.

**Figure 4:**
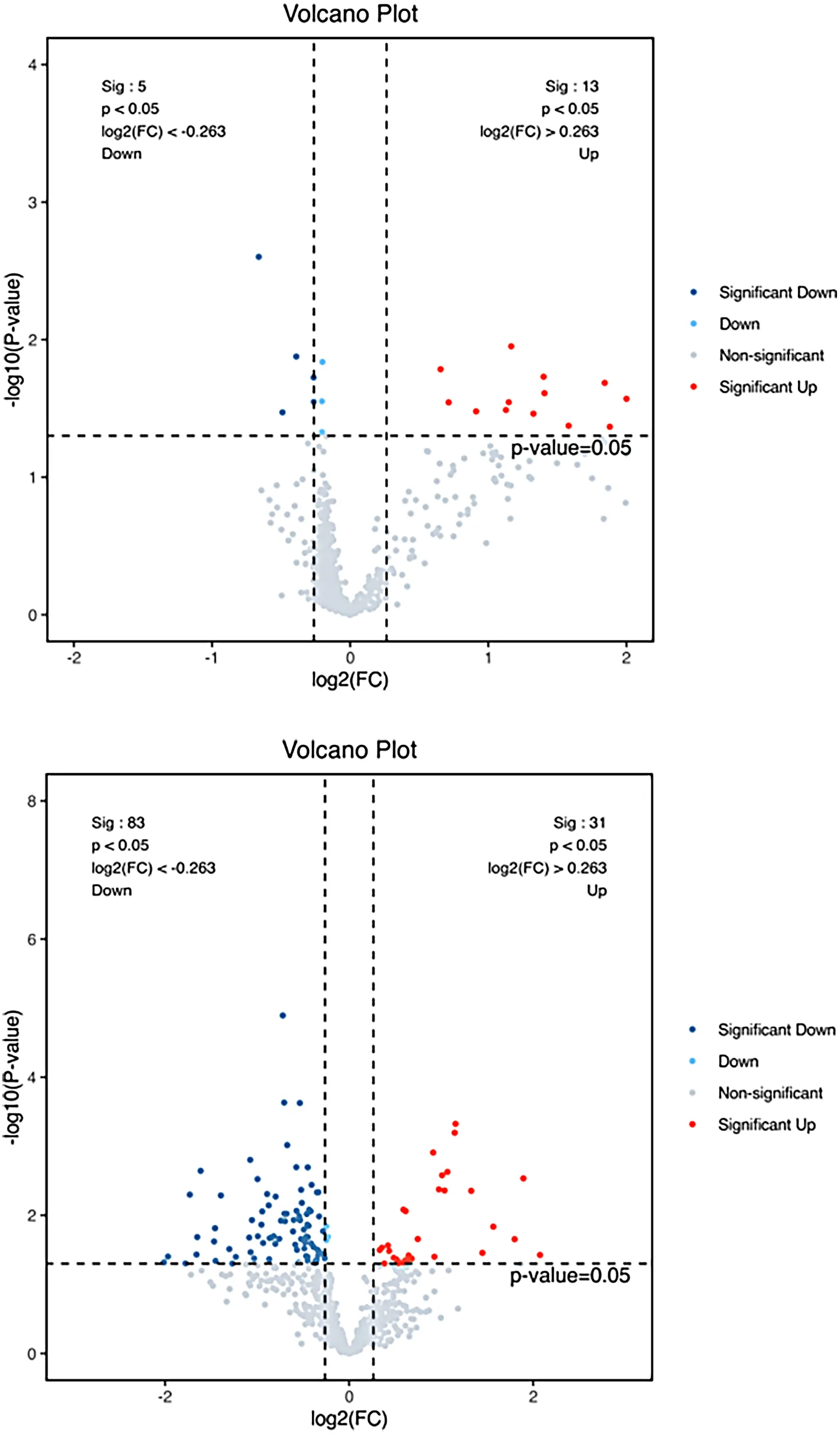
Volcano plots of differentially expressed proteins. OE1 vs. OE2 (left); OE2 vs. NC (right).

#### 3.2.4 Venn analysis

Venn diagram analysis showed that 1 protein was differentially expressed across all three comparisons; OE1-vs.-NC had 146 unique differentially expressed proteins, OE2-vs.-NC had 114, and OE1-vs.-OE2 had 18. OE1-vs.-NC and OE2-vs.-NC shared 73 differentially expressed proteins; OE1-vs.-NC and OE1-vs.-OE2 shared 4; OE2-vs.-NC and OE1-vs.-OE2 shared 9 (Figure 5).

**Figure 5:**
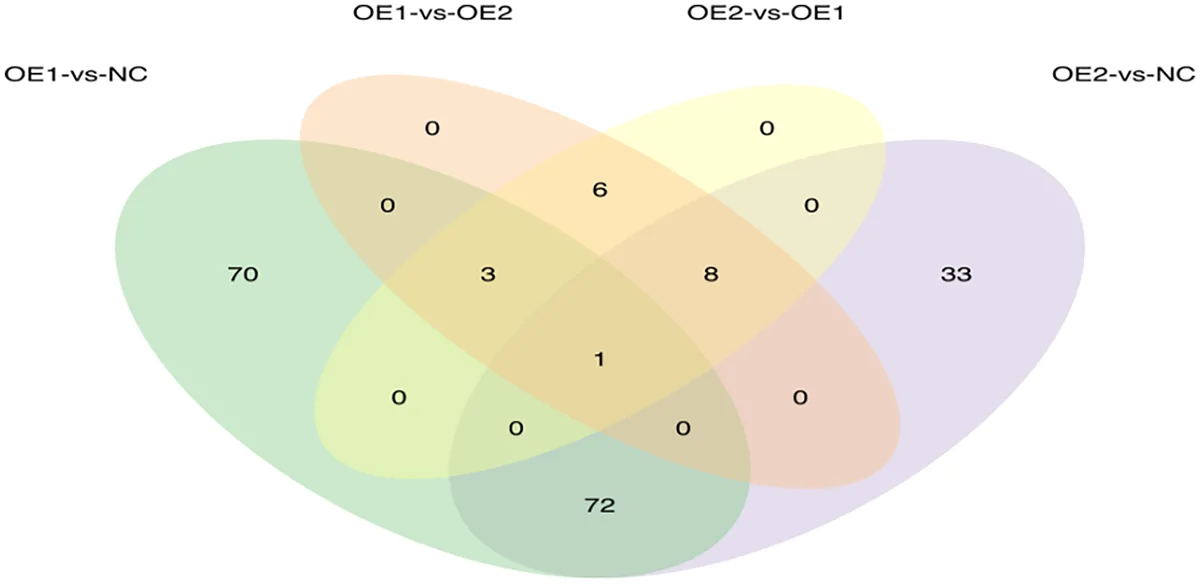
Venn diagram of differentially expressed proteins across comparisons.

#### 3.2.5 Differential protein analysis in PFO patients (OE1 vs. OE2)

Preoperative arterial blood in PFO patients with migraine showed upregulation of inflammation-oxidative stress-related proteins represented by myeloperoxidase (MPO), lysozyme C (LYZ), and heat shock protein 60 (HSPD1), while postoperative arterial blood showed relative upregulation of vascular function-extracellular matrix-related proteins represented by angiotensin-converting enzyme (ACE), collagen alpha-1(VI) chain (COL6A1), and integrin alpha-2 (ITGA2) (Figure 6).

**Figure 6:**
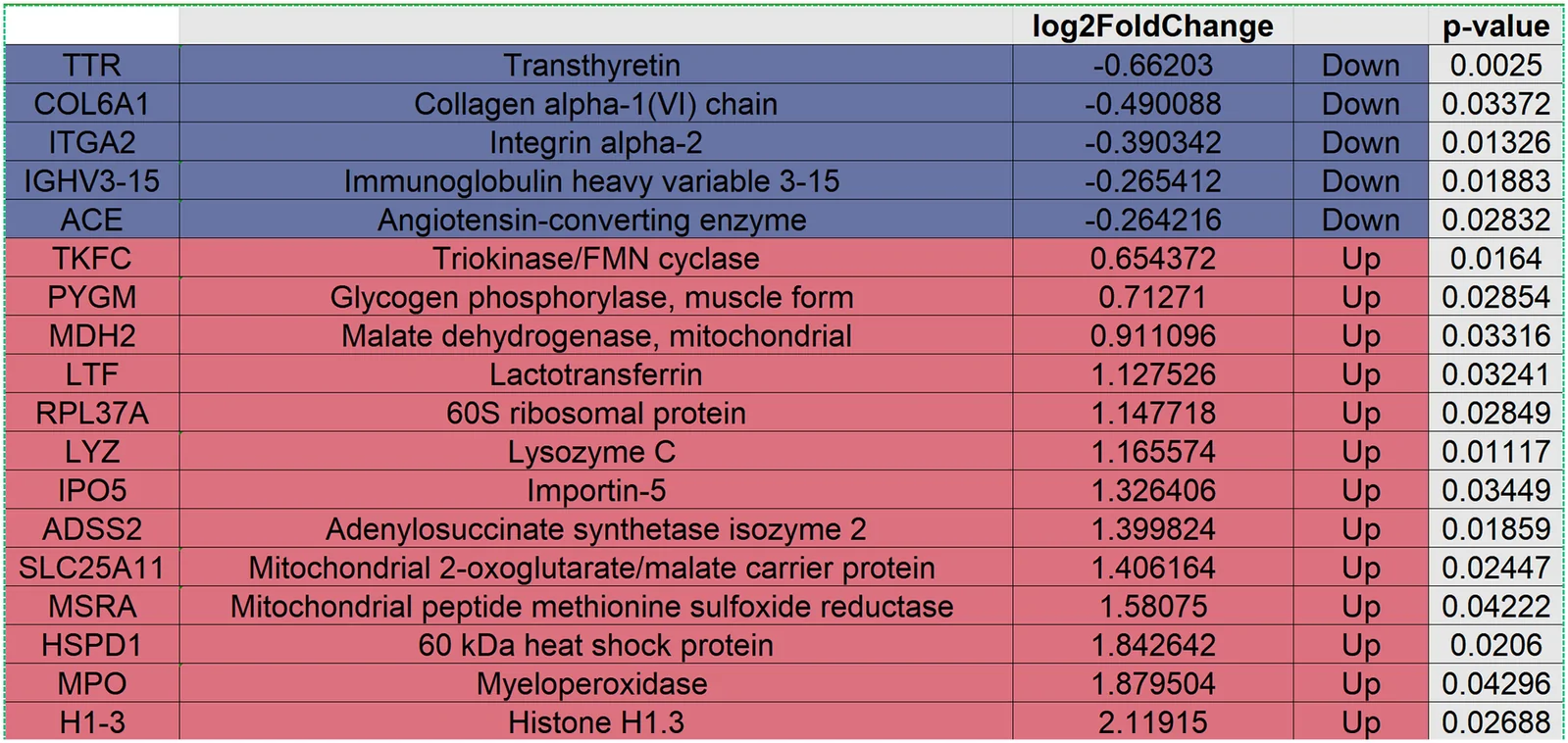
Changes in representative mediators in preoperative and postoperative blood of PFO patients. log₂FC > 0 indicates relative upregulation in OE1 (preoperative); log₂FC < 0 indicates relative upregulation in OE2 (postoperative).

#### 3.2.6 GO functional enrichment analysis of differentially expressed proteins

GO functional enrichment analysis of OE1-vs.-OE2 differentially expressed proteins (*P* < 0.05) revealed:

Biological process: Significant enrichment in “positive regulation of inflammatory response,” “response to oxidative stress,” and “defense response to bacterium,” confirming systemic immune-inflammatory activation and oxidative stress responses in PFO patients with migraine. Enrichment of metabolic terms such as “gluconeogenesis” and “cellular response to amino acid stimulus” suggested that energy metabolism abnormalities also participate in the disease process.

Cellular component: Differentially expressed proteins were mainly localized to “extracellular exosome,” “extracellular space,” “azurophil granule lumen,” and “secretory granule.” Azurophil granules and secretory granules are primarily associated with granule release from immune cells such as neutrophils and macrophages, further supporting the role of immune cell activation in PFO-related migraine.

Molecular function: Differentially expressed proteins were mainly involved in “lipopolysaccharide binding,” “collagen binding,” “heparin binding,” and “metal ion binding.” Lipopolysaccharide binding is a key step in innate immune recognition, and heparin binding is involved in coagulation and inflammatory regulation, both closely related to the pathophysiology of PFO-related RLS (Figure 7).

**Figure 7:**
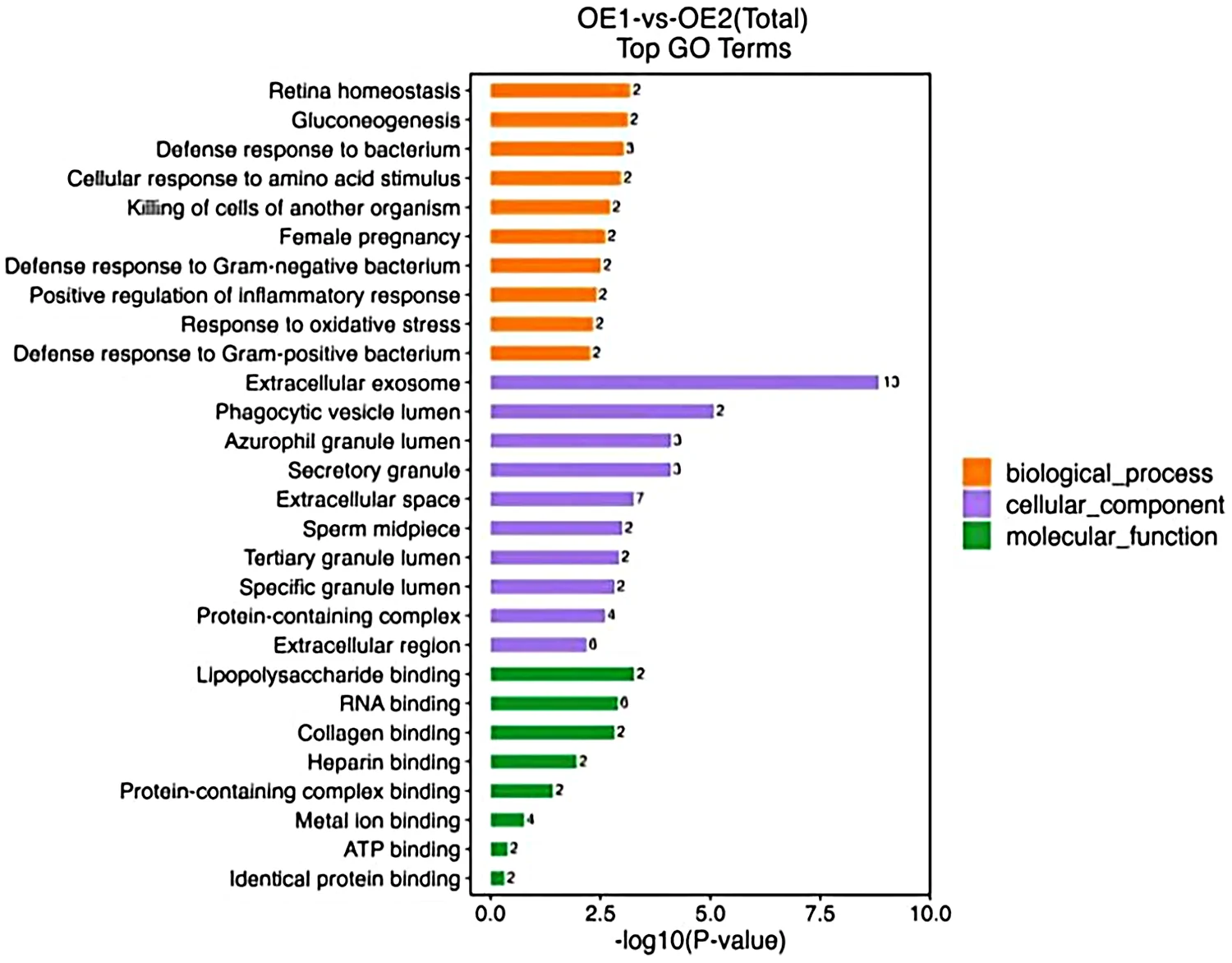
GO functional enrichment analysis of differentially expressed proteins. GO, gene ontology.

#### 3.2.7 Clustering analysis of differentially expressed proteins

Hierarchical clustering analysis showed that OE group and NC group samples could be clearly distinguished at the protein expression profile level, with different expression patterns between the two groups (Figure 8). Key differentially expressed proteins, including P05164 (MPO), P61626 (LYZ), P02766 (TTR), P12821 (ACE), and P10809 (HSPD1), showed differential expression trends in PFO patients with migraine.

**Figure 8:**
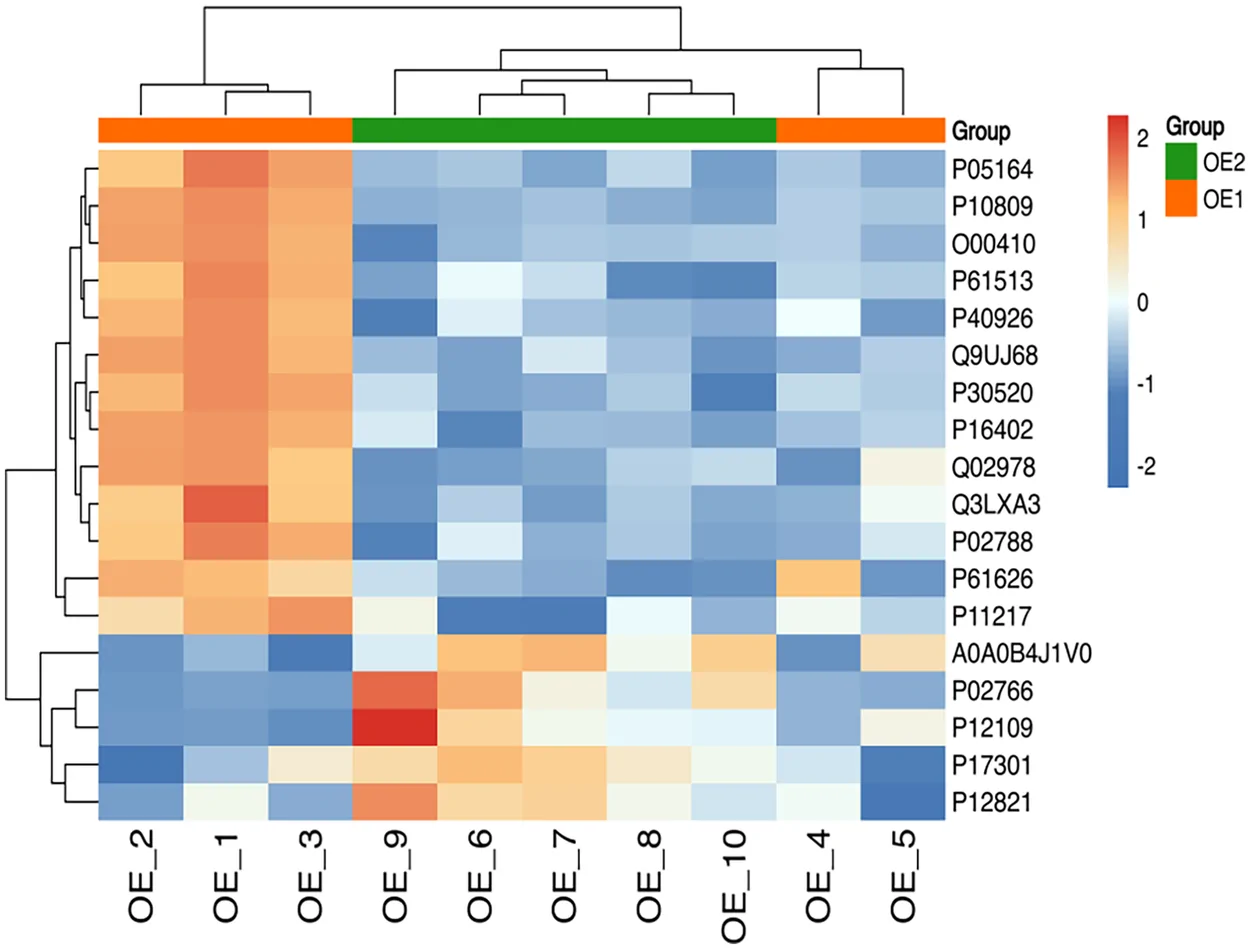
Clustered heatmap.

#### 3.2.8 Differential metabolite analysis

LC-MS-based untargeted metabolomics was used to screen for differential metabolites in serum between PFO patients with migraine after closure and healthy controls, with thresholds of FC ≥ 2.0 or FC ≤ 0.5 and *P* < 0.05.

Compared with healthy controls, patients with PFO and migraine after closure showed differences in the following serum metabolites: dopamine, L-proline, L-tyrosine, D-proline, acetylcarnitine, and galactitol. KEGG pathway analysis showed enrichment in protein digestion and absorption, metabolic pathways, ABC transporter pathways, and arginine and proline metabolism (Figure 9).

**Figure 9:**
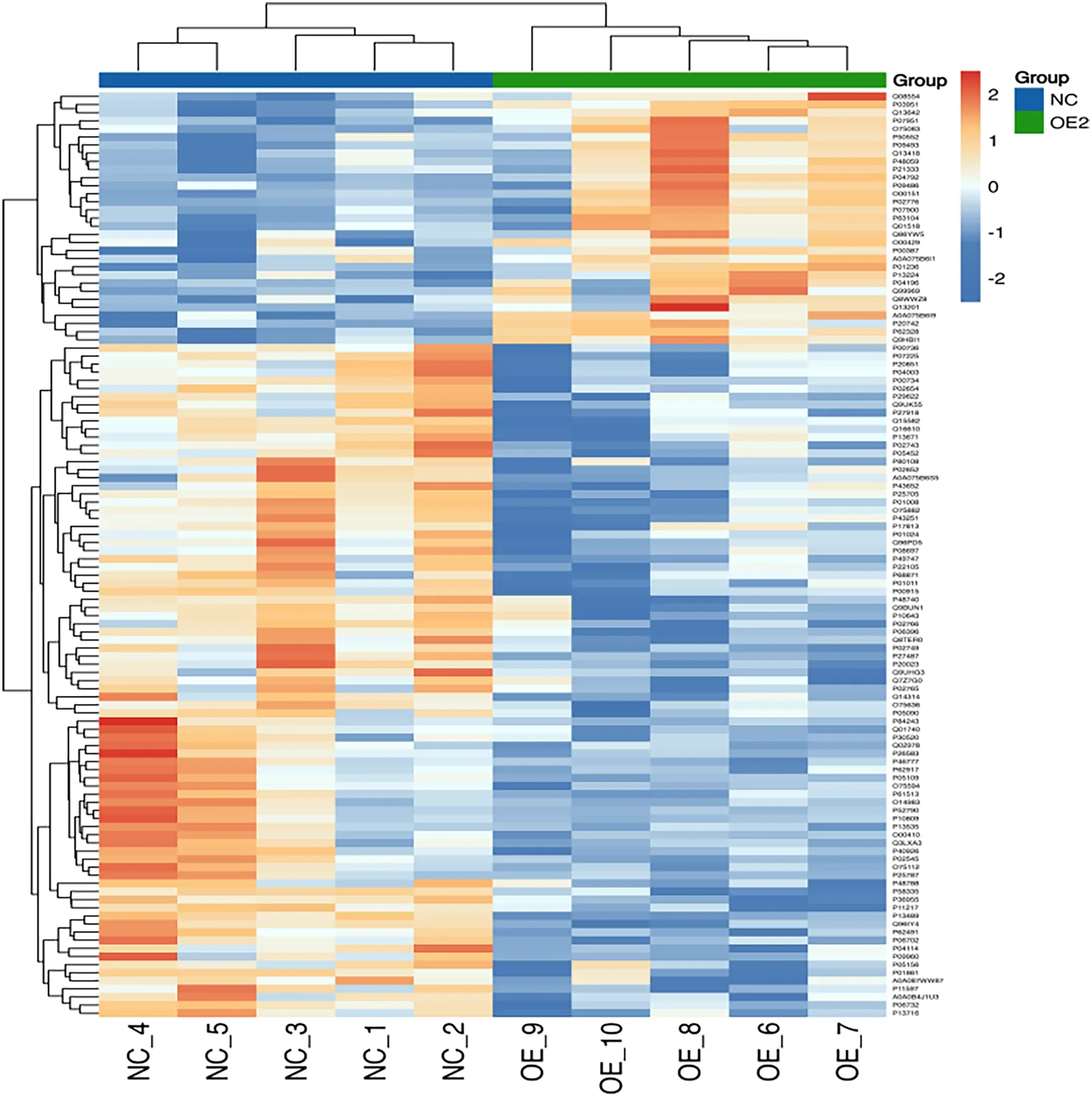
Clustered heatmap.

## 4 Discussion

This single-center retrospective cohort study systematically evaluated the clinical efficacy of transcatheter PFO closure in patients with PFO and migraine and, combined with plasma proteomics, preliminarily explored the potential molecular mechanisms of PFO-related migraine. The results showed: (1) PFO closure reduced VAS, HIT-6, MIDAS, and MSQ v2.1 scores, with improvements greater than in the medical therapy group (*P* < 0.003), and an overall effective rate of 94.83% at 12 months, superior to the medical therapy group (73.47%, *P* = 0.003); (2) PaO₂ and SaO₂ improved after closure; (3) Proteomics revealed differences in protein expression profiles between preoperative and postoperative arterial blood and between patients and healthy controls, with differentially expressed proteins mainly enriched in inflammation, oxidative stress, immune response, and metabolic regulation. These findings provide preliminary molecular evidence for the mechanisms linking PFO and migraine.

### 4.1 Clinical efficacy of PFO closure and comparison with previous studies

In the present study, the overall effective rate at 12 months postoperatively was 94.83% in the observation group, ly higher than 73.47% in the control group. This efficacy exceeds that of several previous randomized controlled trials (MIST, PRIMA, and PREMIUM), which likely failed to meet their primary endpoints due to overly broad inclusion criteria rather than lack of treatment effect, as highlighted by a recent CONSORT assessment (15, 16). Our findings are consistent with recent Asian cohorts reporting 86.7% and 81% improvement rates, respectively, and confirming preferential benefit in patients with migraine with aura and larger shunts (8, 17–20). Notably, the control group also achieved a 73.47% effective rate, suggesting that migraine attacks are influenced by multiple factors and that PFO closure is not the sole determining factor—reinforcing the importance of precise patient selection (21).

In terms of safety, no serious complications (cardiac tamponade, device migration, thrombosis) occurred in this cohort of 58 patients. New-onset atrial fibrillation occurred in 2 patients (3.4%), both with spontaneous reversion, a rate comparable to the 2%–5% reported in the literature (22–24), confirming that transcatheter PFO closure is safe and feasible when indications are strictly adhered to.

Unlike indirect selection strategies such as antithrombotic response, our proteomic approach directly links closure to downregulation of inflammation-related proteins (MPO, LYZ, HSPD1), providing a mechanistic basis for patient selection and distinguishing our study from prior clinical trials that lacked molecular stratification (11, 13, 14, 25).

### 4.2 The special relationship between PFO and migraine with aura

In this study, 66% of enrolled patients were diagnosed with migraine with aura, twice the proportion of those without aura, whereas in the general population, the prevalence of migraine with aura is approximately half that of migraine without aura (26, 27). This disproportionate representation is likely attributable to our inclusion criteria requiring significant RLS, which is more commonly observed in patients with migraine with aura. The preferential association between PFO and migraine with aura be explained by the fact that RLS-induced microemboli or vasoactive substances are more prone to trigger cortical spreading depression—the putative substrate of aura—in susceptible individuals (8, 19). The overrepresentation of this subgroup in our cohort (66%) supports this hypothesis and, if confirmed by future studies, guide more precise patient selection for PFO closure. Further investigation is warranted to elucidate the specific relationship between PFO and aura generation, as well as the underlying genetic and clinical determinants that modulate individual susceptibility.

### 4.3 Potential mechanisms of PFO-related migraine: from hemodynamics to molecular level

The mechanisms linking PFO and migraine have long been debated (14, 28). Our proteomic findings provide a preliminary molecular perspective: preoperative arterial blood showed upregulation of inflammation-oxidative stress proteins (MPO, LYZ, HSPD1), while postoperative blood showed relative upregulation of vascular-ECM proteins (ACE, COL6A1, ITGA2). This differential pattern be tentatively explained by RLS-induced intermittent hypoxia resembling ischemia-reperfusion injury, which triggers mitochondrial ROS bursts, activates NF-*κ*B signaling, recruits neutrophils with MPO release, and upregulates stress proteins (LYZ, HSPD1). Simultaneously, deoxygenated venous blood can induce VEGF via HIF-1*α*, promoting vascular and ECM remodeling (COL6A1, ITGA2). The relative upregulation of vascular-ECM proteins post-closure reflect compensatory hemodynamic responses or device-related endothelialization. Mechanistically, RLS-induced systemic hypoxemia can activate the trigeminovascular system, promote CGRP release, and increase oxidative stress and adhesion molecules in the occipital lobe—closely linked to cortical spreading depression (14, 16, 29, 30).

GO enrichment analysis corroborated these findings, with differentially expressed proteins enriched in “positive regulation of inflammatory response,” “response to oxidative stress,” “azurophil granule lumen,” and “secretory granule”—the latter two associated with neutrophil and macrophage degranulation, supporting immune cell activation in PFO-related migraine. Enrichment of metabolic terms such as “gluconeogenesis” also suggested energy metabolism involvement. PFO closure restore proteomic homeostasis by blocking RLS and correcting hypoxia, thereby alleviating migraine.

### 4.4 Clinical significance of proteomic findings

Although the sample size of the proteomic analysis in this study was limited (only 5 cases), the findings have important implications. First, the differences in protein expression profiles between preoperative and postoperative arterial blood in PFO patients provide a novel molecular perspective for understanding the pathogenesis of PFO-related migraine. Second, the differentially expressed proteins were mainly enriched in inflammation-immunity-oxidative stress pathways, highly consistent with the “RLS → systemic hypoxia → systemic inflammation” hypothesis (19, 31, 32). These results collectively suggest the possibility that amino acid metabolism and neurotransmitter metabolism disorders participate in the pathogenesis of PFO-related migraine (33–35).

Notably, protein expression differences persisted between arterial blood of PFO patients with migraine after closure and that of healthy controls, suggesting that although closure improves symptoms, it not completely restore the protein expression profile to healthy control levels. Differential metabolite analysis showed differences in serum dopamine, L-proline, L-tyrosine, and acetylcarnitine between PFO patients with migraine after closure and healthy controls, with KEGG pathway enrichment analysis suggesting abnormalities in arginine and proline metabolism and ABC transporter pathways. Whether these metabolic differences represent etiological factors of PFO-related migraine, disease sequelae, or are related to perioperative medications (such as antiplatelet agents) or dietary status remains unclear. Additionally, small sample size, interindividual variability, and relatively short postoperative follow-up all affect the stability of the metabolomic results. Further validation through expanded sample sizes, extended follow-up, and animal models for causal inference is needed to clarify the specific associations between these metabolite changes and migraine relief.

### 4.5 Study limitations

This study has several limitations. First, this was a single-center retrospective cohort study with inherent selection bias (observation group: moderate-to-large RLS; control group: moderate-to-small RLS). Although multivariate regression was performed, residual confounding cannot be excluded. Second, the proteomic analysis included only 5 cases per group, rendering findings exploratory. Third, the age ranges of the two groups differed (observation 18–75 years, control 18–60 years). Fourth, the 12-month follow-up rate was 86.9% with self-reported headache data, introducing potential bias. Fifth, long-term imaging follow-up was not systematically performed. Sixth, not all healthy controls underwent TEE to exclude PFO, potentially affecting proteomic specificity. Seventh, the persistent protein differences between postoperative patients and controls reflect the short 1-month follow-up or device-related microenvironmental effects. Eighth, cTCD was not systematically incorporated into outcome stratification.

While these findings are not sufficient to modify current clinical practice, they provide a rationale for future studies.

#### 4.5.1 Knowledge gaps

Key unanswered questions remain: (1) Have the candidate biomarkers (MPO, LYZ, HSPD1) been validated independently?—No. Their predictive value is unknown. (2) What is the optimal timing for PFO closure?—Unknown. (3) Are clinical and proteomic changes durable beyond 12 months?—Uncertain. (4) Does residual shunt correlate with symptom recurrence?—Unassessed. (5) Are these findings generalizable to non-Asian populations, patients without aura, or those with small RLS?—Unknown. (6) Is the inflammatory pathway cause or consequence?—Unresolved. (7) Is PFO closure cost-effective compared with CGRP monoclonal antibodies?—Unevaluated.

#### 4.5.2 Future directions

To address these gaps, we propose: (1) large-scale multicenter prospective cohorts to validate candidate biomarkers; (2) a biomarker-stratified RCT focusing on migraine with aura and moderate-to-large RLS; (3) long-term follow-up (>5 years) to confirm durability and late complications; (4) comparative effectiveness research against CGRP monoclonal antibodies; (5) cost-effectiveness analyses; and (6) mechanistic studies including animal models to establish causality (21, 25, 36).

## 5 Conclusions

First, transcatheter PFO closure improved migraine symptoms in PFO patients, with a 12-month effective rate of 94.83% vs. 73.47% in the medical therapy group (*P* = 0.003), and demonstrated good safety in this cohort.

Second, proteomic findings suggest a potential mechanistic cascade of “PFO → RLS → systemic hypoxia → systemic inflammation → migraine,” supported by: (a) preoperative upregulation and postoperative downregulation of inflammation-oxidative stress proteins (MPO, LYZ, HSPD1) with GO enrichment in inflammatory pathways; and (b) improved arterial oxygenation (PaO₂ and SaO₂) after closure. These findings are exploratory, and causal inference cannot be established from this observational study. Validation in larger prospective cohorts and RCTs is needed before PFO closure can be recommended as a standard treatment.

## Data Availability

The original contributions presented in the study are included in the article/Supplementary Material, further inquiries can be directed to the corresponding authors.
